# Exposed Embolic Coils Observed in a 64-Year-Old Male With Head and Neck Cancer Following Transarterial Embolization for Carotid Blowout Syndrome

**DOI:** 10.1155/2024/7925511

**Published:** 2024-05-13

**Authors:** Jia-Zheng Huang, Wei-Chen Lu, Bo-Ching Lee

**Affiliations:** ^1^Departments of Medical Imaging, National Taiwan University Hospital, Taipei, Taiwan; ^2^Department of Oncology, National Taiwan University Hospital Yunlin Branch, Yunlin, Taiwan

**Keywords:** carotid blowout syndrome, head and neck cancer, transarterial embolization

## Abstract

**Background:** Delayed migration and exposure of embolic coils is a rare complication of endovascular therapy for carotid blowout syndrome.

**Methods:** A 64-year-old man with recurrent tongue cancer noticed the presence of foreign body in the malignant wound on the right side of his neck. He had undergone transarterial embolization on his right vertebral artery, right common carotid artery (CCA), and internal carotid artery (ICA) for carotid blowout syndrome 1 month prior. On physical examination, exposed spring-like metallic coils were observed, covered in brownish granulation tissue, at the bottom of the malignant wound. Neck radiograph and computed tomography confirmed the extrusion and migration of the embolic coils.

**Results:** In this case, the patient was managed by transection of the exposed coils at the wound surface with close monitoring.

**Conclusions:** Computed tomography angiography is essential for assessing the condition of the remaining embolic coils. In cases with thrombosed parent arteries, a conservative approach, like the transection of exposed coils, can be employed as part of the management strategy.

## 1. Introduction

Carotid blowout syndrome is a severe complication of the head and neck cancer with a mortality rate of around 50% [1]. The preferred treatment is endovascular due to its high efficacy in achieving hemostasis [2]. For patients with a robust collateral blood supply from the circle of Willis and a low risk of hypoperfusion, coil embolization of the carotid trunk can be an option, with a relatively lower risk of rebleeding compared to covered stents [3, 4]. While embolization of the external carotid artery typically has a high success rate with minimal complications, the complication rate, such as ischemic stroke, can be as high as 23.7% for embolization of carotid trunk [4, 5].

However, other complications, such as delayed migration of coil embolization for carotid blowout syndrome, are rarely reported in the literature. Therefore, we report the case of a carotid blowout syndrome status post coil embolization, which presented with an exposed embolization coil in the malignant wound.

## 2. Case Report

A 64-year-old man with a history of recurrent tongue cancer presented with exposed embolic coils in a malignant wound on his right neck. A decade prior, he had undergone wide excision and right neck lymph node dissection for right tongue cancer. Two years ago, a recurrence was detected in the right tongue base, leading to repeated tumor excision, lymph node dissection, and adjuvant chemoradiotherapy. However, 7 months ago, he developed skin metastasis and recurrent lymphadenopathies in the right neck. Oral uracil-tegafur (UFUR) was initiated as maintenance therapy, but the malignant wound continued to progress, necessitating wound dressing.

**Figure 1:**
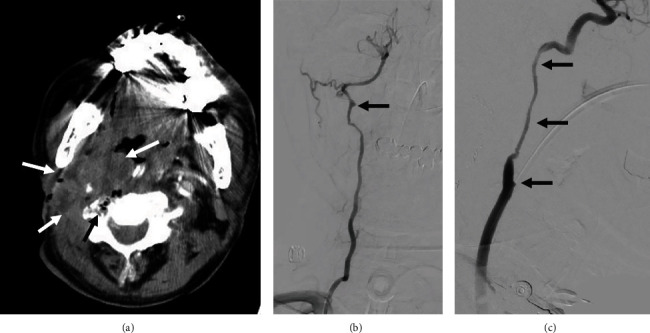
(a) Computed tomography angiography showed encasement of the right common carotid artery, right internal carotid artery, and right vertebral artery by the confluent hypoenhancing tumor (white arrows). Note the presence of air-containing necrosis around the right vertebral artery (black arrow). Angiography of the (b) right vertebral artery and (c) right common carotid artery showed focal vascular irregularity caused by tumor invasion (black arrows).

**Figure 2:**
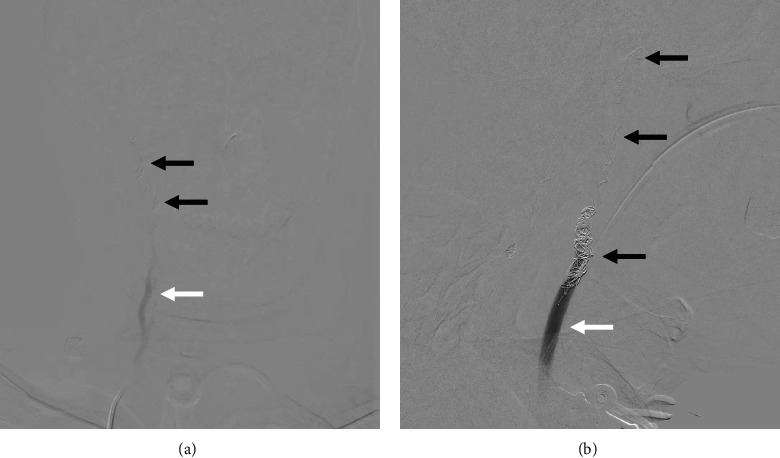
(a) Coil embolization (black arrows) of right vertebral artery was performed until flow stasis (white arrow). (b) Coil embolization (black arrows) of the right internal to common carotid arteries was performed until stasis of antegrade blood flow (white arrow).

One month prior to this presentation, the patient experienced massive bleeding from the malignant wound, unresponsive to compression. Neck radiography and computed tomography angiography (Figure 1(a)) revealed threatened segments of the right vertebral artery, right common carotid artery (CCA), and right internal carotid artery (ICA) within the necrotic tumor. Angiography of these arteries revealed vascular irregularities resulting from tumor invasion (Figures 1(b) and 1(c)), and emergency coil embolization successfully controlled the bleeding (Figure 1(b)) using multiple MicroNester and Tornado pushable coils (up to 18-14-4, Cook Medical, Bloomington, IN) along with two Interlock-Fibered IDC Occlusion Systems (Boston Scientific, Natick, MA) until achieving flow stasis (Figure 2). No ischemic symptoms developed after coil embolization, and no antithrombotic therapy was administered. Afterward, this patient underwent targeted therapy for his head and neck cancer with nivolumab and afatinib. No radiotherapy or operation was performed during this period.

**Figure 3:**
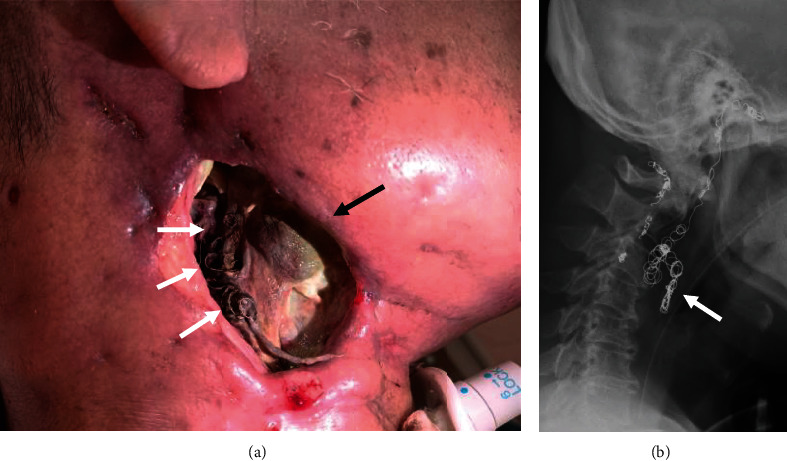
(a) Exposed embolization coils (white arrows) in the malignant wound (black arrow). (b) Neck radiograph showed exposed and migration of embolization coils (white arrow).

**Figure 4:**
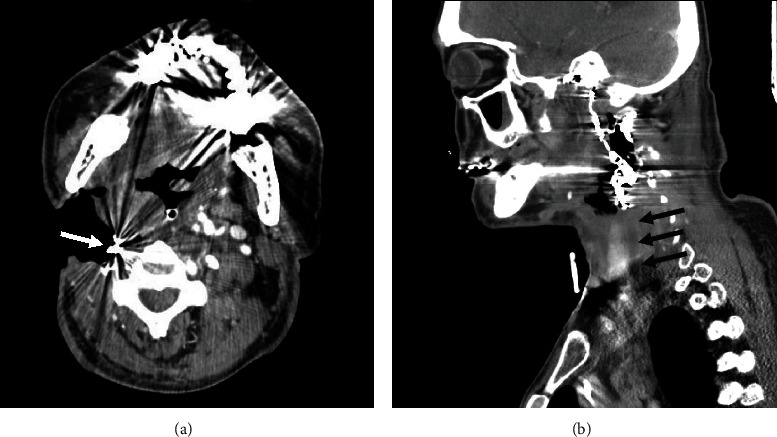
Computed tomography angiography showed (a) exposed embolization coils (white arrows) in the malignant wound of right neck and (b) thrombosed right common carotid artery (black arrows).

At this visit, the patient reported observing a coil-like metallic wire covered with brown granulation tissue at the wound's base. Neck X-rays confirmed the exposure and migration of embolization coils (Figure 3). Since there were still sufficient coils within the thrombosed lumen of the right CCA and right ICA on computed tomography angiography, conservative management by transection of the exposed coils was performed (Figure 4). During his 3-month hospitalization for treatment of malignant wound infection and pneumonia, no further episodes of massive bleeding occurred.

## 3. Discussion

This case emphasizes the occurrence of delayed exposure and migration of embolization coils, which is a rare complication of embolization in the carotid blowout syndrome [6]. Anoxia and necrosis are more prevalent and severe in head and neck cancer compared to many other types of malignancies [7], although the exact pathomechanism remains unclear. The presence of air-containing necrosis or abscess in patients with head and neck cancer serves as an important prognostic factor for patient survival and rebleeding [8, 9]. In this case, some air-containing necrosis was already evident on preprocedural CT before embolization, indicating its necrotic nature. Subsequently, prominent tumor necrosis and cavitation occurred following treatment of carotid blowout syndrome and antitumoral therapy, leading to exposure of the embolic coils. Since coils may become entangled with each other, we suggest to avoid directly pulling out the exposed coils until computed tomography angiography is performed, as this could potentially cause the displacement of other unaffected coils. When there are enough remaining embolic coils in the embolized artery and the lumen is thrombosed, a conservative management approach can be taken, as demonstrated in this case. If there is a high risk of recurrent carotid blowout syndrome due to insufficient intraluminal embolization coils, repeated embolization can be considered.

## Data Availability

The data that support the findings of this study are available from the corresponding author upon reasonable request.
